# The crosstalk between parenchymal cells and macrophages: A keeper of tissue homeostasis

**DOI:** 10.3389/fimmu.2022.1050188

**Published:** 2022-11-24

**Authors:** Yusi Chen, Li Tang

**Affiliations:** State Key Laboratory of Proteomics, National Center for Protein Sciences, Beijing Proteome Research Center, Beijing Institute of Lifeomics, Beijing, China

**Keywords:** parenchymal cells, tissue-resident macrophages, cellular crosstalk, tissue-specific function, mammalian, tissue homeostasis

## Abstract

Non-parenchymal cells (NPCs) and parenchymal cells (PCs) collectively perform tissue-specific functions. PCs play significant roles and continuously adjust the intrinsic functions and metabolism of organs. Tissue-resident macrophages (TRMs) are crucial members of native NPCs in tissues and are essential for immune defense, tissue repair and development, and homeostasis maintenance. As a plastic-phenotypic and prevalent cluster of NPCs, TRMs dynamically assist PCs in functioning by producing cytokines, inflammatory and anti-inflammatory signals, growth factors, and proteolytic enzymes. Furthermore, the PCs of tissues modulate the functional activity and polarization of TRMs. Dysregulation of the PC‐TRM crosstalk axis profoundly impacts many essential physiological functions, including synaptogenesis, gastrointestinal motility and secretion, cardiac pulsation, gas exchange, blood filtration, and metabolic homeostasis. This review focuses on the PC‐TRM crosstalk in mammalian vital tissues, along with their interactions with tissue homeostasis maintenance and disorders. Thus, this review highlights the fundamental biological significance of the regulatory network of PC‐TRM in tissue homeostasis.

## Introduction

Tissues can be considered a collection of cell clusters and intercellular substances. The communication between non-parenchymal cells (NPCs) and parenchymal cells (PCs) creates different organ functions. As basic cellular units, PCs play significant roles and continuously regulate the intrinsic functions and metabolism of tissues (1–4). Hepatocytes account for approximately 60% of total liver cells and 80% of liver tissue volume and perform a series of metabolic functions in the liver (2). Cardiomyocytes account for about 30% of whole cardiac cells in the heart, which drive cardiac contraction and relaxation (3). Alveoli are the functional units of the lungs performing gas exchange (4). The alveolar wall, the main structure of the alveoli, is composed of specialized alveolar type I cells that provide an extensive surface area for gas exchange with the surrounding capillaries and specialized alveolar type II cells that secrete surfactants and other proteins (4). These specialized PCs support the liver in orchestrating systemic metabolism, the heart in regulating blood circulation, and the lung in exchanging carbon dioxide and oxygen. Therefore, they are the primary cells responsible for the organ’s primary function and are essential to tissue homeostasis and systemic physiological processes.

In addition to PCs, NPCs are another large group of cells that make up tissues, such as hepatic stellate cells and Kupffer cells in the liver, macrophages and lymphocytes in the spleen, alveolar macrophages and monocytes in the lung, and glial cells in the CNS. NPCs act as mechanical scaffolds to guide parenchymal repair and regeneration, maintain substance metabolism and nutrition balance, regulate transmitter function, and participate in the immune response (2, 5–7). For example, hepatocytes perform the primary metabolic functions in the liver, whereas NPCs serve regulatory functions, such as pathogen clearance, apoptotic cell phagocytosis, and cytokine secretion (8).

These tissue elements arrange and interconnect to form a particular tissue (9). In tissue, the cell‐cell crosstalk develops a “mutualistic” relationship and produces a specific function output (9). Regarding the organism as an ecosystem, the circulation of matter and energy flow is relatively stable under steady-state conditions. In the short term, the out-of-balance fluctuations can be self-corrected to maintain relative stability. Nevertheless, the regulatory balance of homeostasis can be exhausted in the long term (9, 10). Moreover, certain specific cells will be recruited to generate proper signals and bring the fluctuations back to equilibrium (9–11).

Homeostatic regulation operates based on negative feedback mechanisms that correct deviations of the system state variables from the desired range or setpoint values. When variations are over-large, homeostatic mechanisms are insufficient to maintain system stability. In such cases, inflammatory signals complement homeostatic regulation and enforce the return to homeostasis (9, 10). As a plastic-phenotypic cluster of NPCs, macrophages dynamically participate in signal communication with surrounding cells by producing cytokines, inflammatory and anti-inflammatory signals, growth factors, and proteolytic enzymes (1). Tissue-resident macrophage (TRM) populations stem from yolk sac-derived erythromyeloid progenitors (YS-EMPs) or fetal liver monocytes, which self-renew and proliferate in the steady state (10–12), whereas the niche of TRMs can be replaced with the macrophages generated from bone marrow-derived monocytes (BM-monocytes) in a non-steady state. Some TRMs, such as intestinal macrophages, can be gradually supplemented by Ly6C^hi^ monocyte-derived macrophages during development (10–12). Over the years, the regulation of macrophages and PCs has gradually attracted increasing attention. Growing research has demonstrated that disrupting the balance of macrophage pools triggers tissue homeostasis and development (13–18).

This review briefly summarizes the phenotypes and functions of TRMs in seven organs, focusing on communication with PCs in steady and non-steady states, and discusses how their crosstalk maintains organ homeostasis. Exploring the relationship between PCs and TRMs in homeostasis maintenance may increase our understanding of the formation of non-homeostatic conditions.

## Microglia and neurons coordinate CNS homeostasis

In the CNS, embryonic yolk-sac progenitors generate erythro-myeloid progenitors (EMPs, c-Kit^+^ CD45^+^ CX3CR1^–^ CSF1R^+^ F4/80^–^) and subsequently differentiate into embryonic microglia (11, 19–22). Microglia (CD45^low/int^ F4/80^low/int^ CX3CR1^+^ CD11b^+^) are the first line of defense against infections in the CNS (23). In addition, microglia also contribute to CNS development and homeostasis, such as apoptotic neuron phagocytosis, neuron development, vasculature development, and neuronal circuit formation (24–26). Under physiological conditions, microglia are in a resting state and on standby (25). However, “resting” microglia exist in a process-bearing and ramified phenotype, progressing toward and actively engulfing synapses (“synaptic pruning”) to control their number and maintain proper neuronal functions (27, 28). Additionally, microglia regulate programmed cell death, axon fasciculation, neurite formation, and synaptogenesis (29, 30).

**Figure 1:**
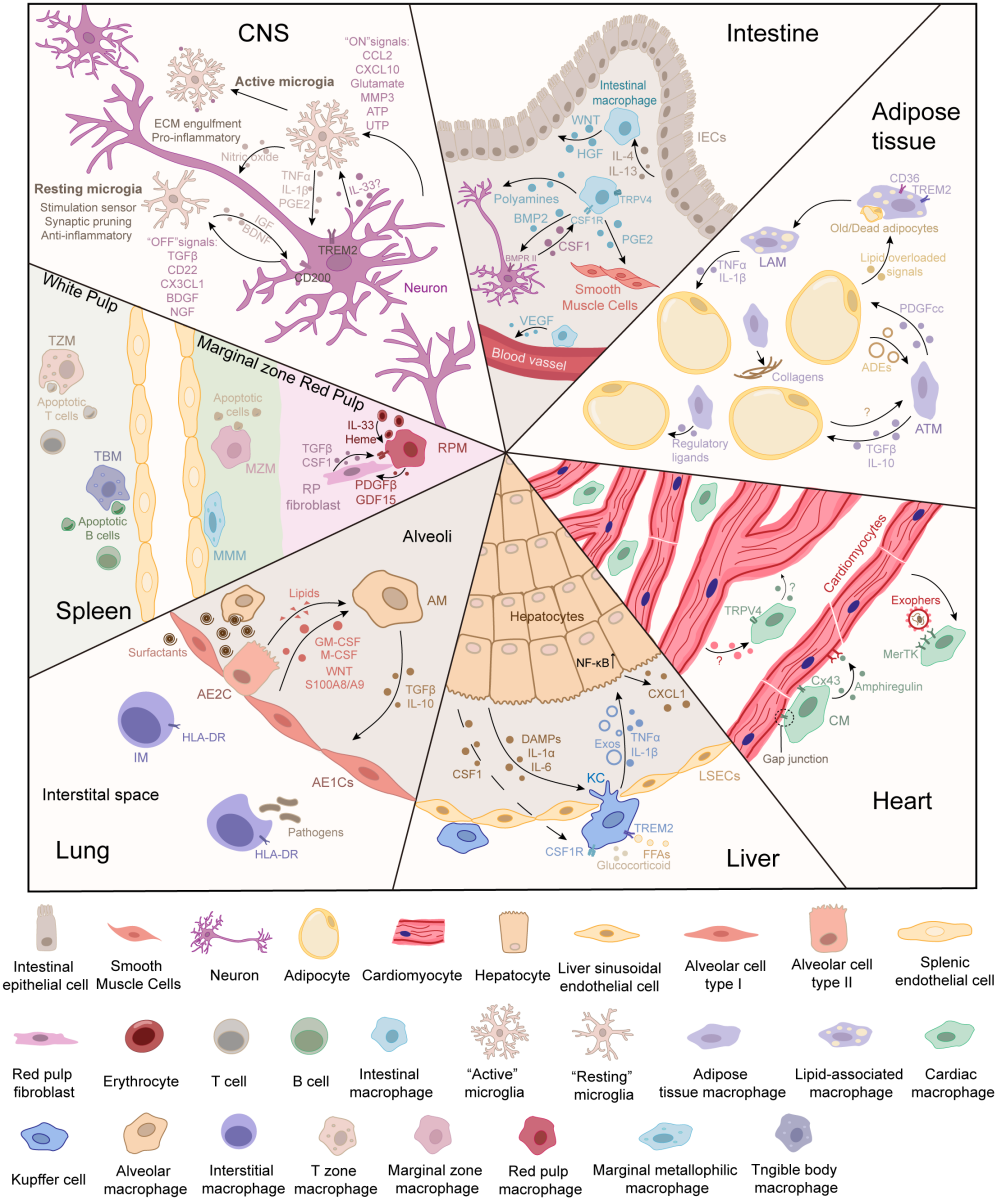
Cell communication between tissue-resident macrophages and parenchymal cells in different tissues. The parenchymal cells can be deemed the “primary cells,” which are responsible for performing the primary function of the tissue. During functions, parenchymal cells can release signals to inform the demand for or the accumulation of metabolites. Tissue-resident macrophages can be deemed “supportive cells,” which sense signals from the environment and parenchymal cells. In turn, tissue-resident macrophages respond to cell demand or modify the microenvironment to maintain the normal physiological functions of primary cells. The crosstalk between tissue-resident macrophages and parenchymal cells maintains tissue homeostasis.

The signal communication between microglia and neurons greatly depends on microglial signaling molecules (31–34). Neurons talk to microglia through “off” and “on” signals, respectively (**Figure 1**) (35). The “off” signals include neurotransmitters, neurotrophins, and transforming growth factor β (TGFβ), which can keep microglia quiescent. The “on” signals include glutamate, chemokines, purines, and triggering receptors expressed on myeloid cells 2 (TREM2) that may be induced by inflammation (35). These signals activate microglia toward a beneficial or detrimental phenotype to regulate neurons under pathological conditions (35–38).

Moreover, microglia sense and catabolize neuron-derived extracellular ATP during neuronal activation (39, 40). This activates microglia in a region-specific manner, leading to the suppression of neuronal activity (39, 40). Interleukin (IL)-33 is a member of the IL-1 cytokine family that is generally secreted into the nucleus. It can activate nuclear factor κB (NF-κB) signaling in target cells after being released into the extracellular space (41). During the early stages of postnatal synaptic maturation, the expression of IL-33 is increased only in the spinal and thalamic astrocytes of the gray matter (42). In the adult brain, IL-33 is widely expressed in the corpus callosum, hippocampus, thalamus, granular layer, and cerebellum white matter (43). Recent research suggests that microglia engulf the extracellular matrix (ECM) under the regulation of neuronal IL-33 in the adult hippocampus (43).

Microglia maintain a dynamic relationship with neurons. The “quiescent” microglia are more like “nannies” that take care of the growth and development of neurons. When neurons encounter stressful conditions, microglia become “fighters” against the hostile environment. Sometimes the weapons of these warriors may accidentally injure innocent victims. To confront pathogen invasion, microglia are activated and release several bioactive molecules to strike down pathogenic bacteria (44–46). These active molecules from amoeboid-like microglia may mis-strike the healthy neurons. The extracellular ATP released by neurons during neuronal activation is sensed and catabolized by microglia (39). This activates microglia in a highly region-specific manner, leading to the suppression of neuronal activity (39). In brief, ATP promotes the recruitment of microglial protrusions whereas the microglial ectoenzyme CD39 hydrolyzes ATP into AMP (40). AMP is converted into adenosine by CD73 and subsequently suppresses neuronal responses (40). Additionally, the “resting” microglia prevent sympathetic overactivation by maintaining Kv4.3 (a potassium channel) on presympathetic neurons (47).

Acting as a “double-edged sword,” microglia play a pivotal role in maintaining tissue homeostasis while partially promoting neurological disease development when exposed to external and internal insults (36, 48, 49). A release of diverse nucleotides accompanies nerve injury, and some of these nucleotides act as “find/eat-me” signals in mediating neuron-glial interplay (46). As mentioned above, the nucleotides ATP and ADP are predominant signal transmitters in mechanical stimulation-induced intercellular Ca^2+^ wave (ICW) communication by acting on P2Y12/13 receptors in BV-2 microglia (46). Once microglia are activated, they participate in developing, spreading, and potentiating low-grade neuroinflammation (50). The inflammatory-activated glial cells exhibit cellular changes that alter their communication with each other and neurons and render neurons more excitable (50). Thus, pain transmission is enhanced and prolonged (50). In neurodegenerative diseases, such as amyotrophic lateral sclerosis (ALS) and Alzheimer’s disease (AD), a unique population of microglia is termed disease-associated microglia (DAM) (21). Similar to DAM, aged microglia exhibit an elevated expression of transcripts upregulated in neurodegenerative diseases, including *Cxcr4*, *Clec7a*, *Axl*, *Lgals3*, and *MHC-II*, which are linked to neuronal loss and exacerbation of the disease (51, 52).

Overall, strategic communication exists in the microglia–neuron axis in physiological and pathological states. However, how dysfunctional microglia–neuronal communication affects disease progression or onset at various stages is still unclear. Using the high-dimensional techniques, a map of microglial diversity has been described on a temporal and spatial axis (24, 53–55). Thus, it is better to use various research methods to comprehensively understand the multi-omics states of condition-specific microglia, which may be essential for understanding the physiological heterogeneity of microglia–neuron interactions and controlling CNS diseases.

## Crosstalk between macrophages and epithelial cells maintains gastrointestinal motility and secretion

The gastrointestinal (GI) tract, consisting of the small intestine (SI) and colon, is the center of nutrient digestion. Intestinal macrophages are an abundant immune member in the gut and play a crucial role as function keepers and adjusters (56, 57). Different from Kupffer cells and microglia, intestinal resident macrophages (CD45^+^ F4/80^+^ CD64^+^ CX3CR1^hi/int^ CD11b^+^) are derived from fetal liver monocytes and gradually become supplemented by Ly6C^hi^ monocytes during development (57–59). They maintain self-renewal and reside mainly in the lamina propria (LP macrophages, CD11c^+^ CD14^+^) and muscularis (muscularis macrophages, CD11c^lo^ CD4^+^ TIMD4^+^ RELMα^+^) (**Figure 1**) (57–59).

Gastrointestinal secretion is essential for the movement and absorption of nutrients and ions across intestinal epithelial cells (IECs) (60). Intestinal macrophages significantly influence epithelial integrity and mucosal permeability by secreting cytokines to IECs and submucosal neurons (60). A recent study demonstrated that monocyte-derived PTGER4^+^ intestinal macrophages promote the healing and repair of the intestinal mucosa by CXCL1 secretion (61). Moreover, macrophages found in the submucosa can maintain the integrity of the submucosal vasculature (62, 63). The intestinal macrophages at the distal colon protrude into the epithelium *via* balloon-like protrusions that prevent the absorption of fungal toxins to preserve mucosal integrity (63).

Gut motility is potentially modulated by crosstalk among enteric neurons, intestinal macrophages, and smooth muscle cells (64). For example, the interaction between muscularis macrophages and intestinal smooth muscle cells is mediated by TRPV4 channels (65). After activating TRPV4 signaling, muscularis macrophages release prostaglandin E2 (PGE2), which directly activates intestinal smooth muscle cells to trigger muscle contraction in a paracrine manner (65). In addition, a subset of intestinal macrophages that reside in the lamina propria (LP) is responsible for the clearance of apoptotic and senescent epithelial cells (66). They promote epithelial integrity by expressing metalloproteinases and cytokines that stimulate tissue remodeling and the renewal of epithelial stem cells, such as hepatocyte growth factor (HGF), PGE2, and WNT ligands (66). Moreover, intestinal macrophages and enteric neurons interact through bone morphogenetic protein (BMP) and colony-stimulating factor 1 (CSF1). Previous research has shown that macrophage-derived BMP2 promotes neuronal activity (67). Reciprocally, enteric neurons can maintain the self-renewal capability of intestinal macrophages through CSF1 (64). Then, local macrophages can adjust intestinal muscle contraction by inducing the production of neurotransmitters, thereby controlling peristalsis (67).

The unique environment of the GI tract is likely to shape the heterogeneity of intestinal macrophages (both resident and recruited macrophages) (56). However, except for immune functions, how the spatial molecular communication of intestinal macrophages senses signals and regulates IECs or other cells to coordinate gut activity remains unclear. How intestinal macrophage subsets play heterogeneous roles in various gastrointestinal diseases also remains unknown. Several chronic inflammatory conditions affect the GI tract and are referred to as inflammatory bowel disease (IBD) (68, 69). IBD is characterized by recurrent bouts of inflammation in the GI tract (68, 69). Endogenous damage-associated molecular patterns (DAMPs) released from injured intestinal epithelial cells activate intestinal macrophages to release abnormal inflammatory factors, recruit monocytes, and promote their proinflammatory transformation, consequently aggravating inflammation and tissue damage (68, 69). In addition, at the onset of the disease, the intestinal epithelium is damaged and the glial cells and neurons in the enteric ganglia are injured or overactivated, resulting in gastrointestinal motility disorders (69). Nevertheless, depleting mature intestinal macrophages alone can cause the death of intestinal epithelial cells and inflammation (63). How macrophage function is affected by gastrointestinal inflammation remains to be studied in the future.

## Adipose tissue macrophages and adipocytes regulate energy metabolism

Adipose tissues are the primary reservoir for storing energy substrates and have adapted to respond rapidly to caloric fluctuations. According to physiological functions, morphology, characteristics, and localizations, adipose tissues are divided into three types: brown adipose tissue (BAT), beige adipose tissue, and white adipose tissue (WAT) (70–72). Adipocytes are the main site of energy metabolism in adipose tissues, such as energy intake and fatty acid release, which have been intensively studied. In WAT, macrophages comprise 30%–50% of the immune cells (70, 73). Through scRNA-seq analysis, five subpopulations of adipose tissue macrophages (ATMs, CD45^+^ F4/80^+^ CD11b^+^ CD64^+^) have been found, including vascular-associated macrophages (VAMs, CD9^–^ MHC-II^lo^ LYVE1^hi^), lipid-associated macrophages (LAMs, CD9^+^ MHC-II^hi^ LYVE1^lo^), infiltrated monocyte-derived macrophages (CD11b^+^ Ly6C^+^), and two additional minor subpopulations of ATMs (73–75). ATMs are derived from YS-EMPs and BM monocytes and are mainly distributed around adipocytes (76).

ATMs account for 5%–10% of stromal cells in lean adipose tissue (76). The crosstalk between macrophages and adipocytes coordinates the functions of adipose tissues (**Figure 1**) (70). When sensing excessive free fatty acids (FFAs), ATMs facilitate the secretion of PDGFcc to increase lipid storage in white adipocytes (77). PDGFcc blockade redirects unstored lipids in BAT and increases thermogenesis (77). Old adipocytes send out “find-me” and “eat-me” signals, which trigger phagocytosis and IL-6 secretion by macrophages (70, 78, 79). The phenotype of ATMs changes under different conditions. Alternatively activated (M2) ATMs may be predominant in physiological homeostasis, and classically activated (M1) ATMs are increased in conditions of obesity (70). M2 macrophages rebuild the microenvironment and regulate systemic glucose homeostasis *via* TGFβ (80). In addition, M2 ATMs can affect adipocyte thermogenesis, contributing to the regulation of energy storage and ready response to energy demands in WAT (71). M2-derived slit guidance ligand 3 (Slit3) stimulates the release of norepinephrine by binding to the specific receptor in sympathetic neurons, thereby improving adipocyte thermogenesis for cold adaptation (81). In addition to maintaining metabolic homeostasis, ATMs also orchestrate the source of some bona fide adipocytes by promoting the hematopoietic-to-mesenchymal transition (82). CD206^+^ ATMs are predominantly M2 macrophages, and ablation of these ATMs improves systemic insulin sensitivity through TGFβ signaling (81).

However, under conditions of obesity, chemokines secreted by hypertrophic adipocytes recruit large numbers of monocytes that differentiate into ATMs, which account for 40%–50% of the stromal cell population (76). Recruited monocyte-derived ATMs often surround damaged adipocytes and form a crown-like structure (CLS) (83–85). These ATMs clear dead cell debris and lipid droplets and contribute to maintaining the integrity of adipose tissue (76). During CLS formation, adipocyte death locally induces ATM metabolic activation and increased lipid metabolism, which may be involved in meta-inflammation development (86, 87). In hypertrophic adipocytes, monocyte-derived macrophages act as early sensors of metabolic changes and produce tumor necrosis factor α (TNFα) and IL-1β, which mediate hepatosteatosis and insulin resistance (77, 88). Moreover, the signals released from adipose tissues, such as exosomes (adipocyte-derived exosomes, ADEs), adipokines, cytokines, and lipids, can affect peripheral tissues and macrophages in an endocrine manner (77, 89). Interestingly, ADEs carrying Sonic Hedgehog (Shh) promote the development of insulin resistance by stimulating macrophage activation to secrete inflammatory cytokines (89, 90). ADEs also act as carriers of miR-34a, exacerbating obesity-induced systemic inflammation and metabolic dysregulation (89, 90). However, during high-fat-diet (HFD)-induced epididymal white adipose tissue (eWAT) remodeling, ATMs are most closely associated with blood vessels, preventing the dysregulation of ECM composition and the formation of tissue fibrosis (91).

As mentioned above, the regulation of physiological and metabolic homeostasis and the inflammatory response in adipose tissue have been described. However, more detailed molecular mechanisms of the macrophage‐adipocyte crosstalk and their roles in obesity-related diseases still need to be investigated. In addition, how the macrophage populations surrounding the distinct parts of adipose tissue accumulate and function differently is still unclear.

## Kupffer cells collaborate with hepatocytes to contribute to liver homeostasis

The liver is a multitasking organ that assumes diversified functions, such as protein synthesis, lipid metabolism, detoxication, and amino acid metabolism (2). In mice, there are two types of liver macrophages: yolk sac (YS)-derived macrophages and monocyte-derived macrophages (92–95). Specifically, KCs (CX3CR1^–^ TIMD4^+^ CLEC4F^+^) are the only YS-derived macrophages in the liver (93). Self-renewing KCs are distributed along the hepatic sinusoids (**Figure 1**). Hepatic stellate cells (HSCs), hepatocytes (HCs), and endothelial cells (ECs) compose the KC niche and imprint identity (96). It has been reported that stimulatory signals in the tissue environment contribute to hepatic macrophage differentiation (97). During liver development, EMPs occupy most liver niches and are generated in KCs, whose identity and self-renewal are maintained through BMP9/BMP10/ALK1 signaling and *Smad4*-dependent pathways (98–102).

Macrophages play a pivotal role in maintaining immune defense and liver homeostasis. An increasing number of studies have suggested that the crosstalk axis of KC‐HC modulates metabolic homeostasis. Lipid metabolism is a critical functional feature of the liver. During fasting and feeding, the liver regulates lipid fluxes through lipogenic and oxidative pathways to adjust to the altered energy state. In the physiological state, excess lipids are mainly stored by adipose tissue and not the liver. KC-derived IL-1β contributes to suppressing the expression of hepatokines in hepatocytes and lipolysis in adipose tissue (103). This suggests that macrophages can promote the proper storage of excess lipids and play an essential role in liver–adipose tissue communication. TNFα, another proinflammatory cytokine from macrophages, suppresses the nuclear translocation of GR and the ketogenesis pathway in HCs (104). Thus, hepatic ketogenesis is inhibited when the body has enough energy sources during feeding (104). During fasting, macrophage GR suppresses the expression of TNFα (104). The limited production of TNFα promotes the mutual intercellular crosstalk between liver macrophages and HCs, directly influencing glucocorticoid signaling and ketogenesis by reshaping the hepatic transcriptional response to coordinate fasting homeostasis (104). In contrast, HCs generate acetoacetate (AcAc) from fatty acid-derived acetyl-CoA *via* a series of enzymatic reactions (105). AcAc acts as a shuttle between HCs and M2 macrophages (105). These studies suggest that crosstalk between HCs and liver macrophages is related not only to cytokines but also to cellular metabolites. Liver macrophages can produce exosomes containing insulin-sensitizing miR-690 that directly inhibits *de novo* lipogenesis and insulin resistance in HCs through the miR-690–*Nadk* axis (106, 107). However, the accumulation of anti-inflammatory macrophages in the liver may drive insulin resistance by increasing cytokine secretion (108, 109). Additionally, KCs were found to act as central regulators in cholesterol homeostasis. Under iron overload, KCs transfer LDL-derived cholesterol to HCs in an Abca1-dependent manner (110). Moreover, macrophages can synthesize anti-inflammatory fatty acids by activating the LXR signaling pathway and SREBP1 signaling pathway, regulating the functions of surrounding HCs in a paracrine manner (111, 112).

However, KCs may exert dual actions on lipid metabolism in hepatocytes. The FFAs released from adipose tissues promote hepatic triglyceride storage, and fatty acid oxidation is inhibited by KC-derived IL-1β in a PPARα-dependent manner (113). The increased secretion of KC-derived IL-1β promotes hepatocyte damage and the progression of ethanol-induced liver diseases (114). Additionally, pyroptotic hepatocytes release IL-1β to stimulate KCs; in turn, KC-derived proinflammatory signals amplify liver inflammation (115). Moreover, DAMPs are sensed by KCs, leading to the release of KC-secreted tumor necrosis factor α (TNFα) to promote chemokine expression in HCs (116). Under HFD conditions, CD11b^+^ F4/80^+^ macrophage-derived TNFα triggers *Sarm1*-dependent sympathetic neuropathy and insulin insensitivity in HCs (117). Lipid overload in HCs induces lipotoxicity and oxidative stress, resulting in damage to HCs with the concomitant release of DAMPs (118). HC-derived FFAs induce the production of IL-1β mtDNA in KCs (119). Reciprocally, this may aggravate the accumulation of hepatic lipids and fatty degeneration (119, 120).

KCs also play a dual role in the immunocompetent mouse model of acute hepatitis B viral (HBV) infection. The stimulation of KCs with IL-6 or TNFα suppresses the expression of LSECtin and accelerates the clearance of liver adenovirus. In contrast, the activation of IL-4, IL-10, or IFN-g in KCs upregulates LSECtin expression and delays viral clearance (121). Additionally, macrophages engulf apoptotic cells and produce anti-inflammatory/tissue repair factors in an LSECtin-dependent manner in IBD (122). However, LSECtin is upregulated in the liver after HBV infection, implying that KCs are hijacked by HBV and have to protect the liver from inflammation by delaying viral clearance (121).

Briefly, we introduce the signal communications between KCs and HCs in metabolic homeostasis and inflammation. Under non-homeostatic conditions, the hepatic niche occupied by KCs is gradually supplemented by Ly6C^hi^ monocyte-derived macrophages (10–12). The differential functions of KCs and monocyte-derived macrophages in diseases, such as NAFLD, remain unclear. In addition, studies on the crosstalk between KCs and HCs in homeostasis have focused more on the unsteady state than the steady state. There is still plenty to discover about the KC‐HC interaction axis.

## Cardiac macrophages and cardiomyocytes maintain cardiac pulsation and energetics

The heart is composed of four chambers and is a complex and vital organ. The cardiovascular system includes blood vessels and blood and is responsible for transporting nutrients and oxygen throughout the body and removing metabolic wastes in cells. During the steady state, cardiac macrophages (CMs, CD11b^+^ F4/80^+^ MHC-II^high/low^ CD64^+^ MerTK^+^) occupy most of the immune niche in the cardiac interstitium (**Figure 1**) (123, 124). Multiomics and fate-mapping studies revealed that CM subsets can be identified as TLF^+^ CMs, CCR2^+^ CMs, and MHC-II^hi^ CMs (123).

The heart requires the precise regulation of heterogeneous cell populations for intense metabolic and mechanical demands. The complex crosstalk between CMs and cardiomyocytes controls cardiac impulse (125). CMs are closely connected with cardiomyocytes and crucially maintain cardiac impulse conduction through gap junctions supported by *Areg* (coding amphiregulin) (126). During the acute phase of myocardial infarction (MI), leukocytes and monocytes are recruited to the ischemic area by CMs (127). The RNA-seq data of single AV node macrophages show that macrophages have an electrical connection with cardiomyocytes through Cx43-containing gap junctions (16, 128). These results suggest that CMs may significantly contribute to conduction abnormalities. In addition, CMs may impact morphogenesis and development in the cardiac conduction system (129, 130). For example, CM ablation in a *Cd11b^DTR^* mouse induces a progressive atrioventricular block (16). A flow cytometric analysis of *Cx3cr1^GFP/+^* fetal hearts, combined with EGF-like module staining, revealed the active recruitment of macrophages at E12.5-16.5 and proliferation throughout the time course of cardiac development (129).

Similar to TRMs in other tissues, cardiac macrophages have a potent phagocytic capacity to remove necrotic debris that prevents myocardial infarct (MI)-induced arrhythmias (131). Treatment with a CSF1R inhibitor or the depletion of recruited CMs increases post-MI ventricular tachycardia, ventricular fibrillation burden, and myocyte death (131). Nevertheless, as highly specialized cells in the heart, cardiomyocytes contain large numbers of mitochondria and eject redundant mitochondria and other materials in subcellular vesicles to partly solve the intense energetic demand (132). Taken together, resident CMs crucially contribute to cardiac homeostasis maintenance.

However, in cardiac diseases, the TRM niche is occupied by CCR2^+^ monocyte-derived macrophages (131). The recruited CMs may initiate the inflammatory cascade that promotes tissue injury and suppresses tissue repair. Thus, different CM subsets serve different roles that require more precise approaches to understanding their character and functions during pre- and postnatal developmental stages (131). Regarding spatial distribution, the intratissue heterogeneity of CMs has not been clarified (123, 128). The disturbance of CM–cardiomyocyte communication may involve a series of heart diseases, including hypertension, ischemia, arrhythmias, and myocarditis (123). Whether CMs distribute homogeneously and how macrophage phenotypes change during disease progression have not been elucidated.

## The macrophage‐alveolar epithelial cell axis regulates pulmonary functions

The lungs mainly carry out gas exchange in alveoli, which are rich in connective tissues such as capillaries, elastic fibers, mesh fibers, and collagen fibers. There are two kinds of epithelial cells on the alveolar surface: type I alveolar epithelial cells (AE1Cs) and type II alveolar epithelial cells (AE2Cs) (4). To combat foreign pathogens, many immune defenders, primarily macrophages, are located in the lungs (**Figure 1**) (4, 10–12). Pulmonary macrophages phagocytize surfactants, inhaled stimuli/invaders, and apoptotic and fragmented cells to maintain lung homeostasis (133, 134). Different microenvironments shape resident macrophages as distinct populations, such as alveolar macrophages (AMs, CD64^+^ MerTK^+^ F4/80^+^ SiglecF^hi/–^ CD169^+/–^) and interstitial macrophages (IMs, LYVE1^lo/hi^ CD64^+^ MerTK^+^ F4/80^+^ SiglecF^–^) (135). AMs and IMs reside in two anatomical compartments and perform slightly different functions (135, 136). IMs are located near many non-hematopoietic cells (136). In addition, IMs are not as abundant as AMs and have lower phagocytic potential (137, 138). Thus, IMs act as a second line of defense against invaders (137, 138).

AMs are located in the lumen of the alveoli and are surrounded by AE1Cs, AE2Cs, and stromal cells (135, 136). In 1-week-old mice, alveolar epithelial cell-derived granulocyte-macrophage colony-stimulating factor (GM-CSF) provides the instructive cytokine signal for AMs to thrive (139). Mature AMs adhere tightly to the luminal side of alveolar epithelial cells, continuously capturing and phagocytosing large amounts of inhaled pathogens and particles without triggering an influx of neutrophils and excessive inflammation (140). The accumulation of surfactants on the alveolar space increases surface tension and leads to alveolar collapse and respiratory failure (141, 142). AMs are responsible for avoiding unnecessary inflammation and capturing and metabolizing surfactants to maintain the biomechanical homeostasis of the lungs (134, 135, 141). Additionally, AMs contribute to inflammatory-associated or non-inflammatory responses through the macrophage–epithelial cell axis to regulate lung homeostasis (133–136). In rats, AMs transport miR-21-5p to tracheal epithelial cells by exosomes that can promote the epithelial–mesenchymal transition (EMT) (143). In mice, cytokines released by macrophages regulate the transcription factor CEBPB in pulmonary epithelial cells (144).

Conversely, pulmonary epithelial cells also influence macrophages. The epithelium-derived WNT and S100A8/A9 regulate the phenotypes and functions of macrophages (145). *In vitro*, AMs lost the expression of genes involved in adhesion molecules, lipid metabolism, TGFβ signaling, and oxygen response (146). However, when the cultured cells are transferred back into the lungs, the *ex vivo* expanded AMs can reacquire their *in vivo* expression profile and identity (146). These findings suggest a potential role for epithelial cells in the maintenance of the AM phenotype (146).

In the airways of chronic obstructive pulmonary disease (COPD) patients, the accumulated oxidized lipids in pulmonary epithelial cells may reduce the phagocytotic ability of AMs (147). With overloaded phagocytosis, AMs trigger inflammation by producing chemokines and proinflammatory cytokines that recruit and activate neutrophils, further contributing to lung damage and systemic “cytokine storms” (136). Although these macrophages have been extensively studied, there are still numerous questions. The mechanisms of the macrophage‐epithelial cell axis in different diseases and the use of macrophage transplantation as an immunotherapeutic approach still require further investigation.

## Red pulp macrophages and fibroblasts orchestrate splenic homeostasis

As the largest secondary lymphoid organ in the body, the spleen also functions as a blood reservoir and filter and participates in immune defense, iron homeostasis, and cell reservoirs (for red blood cells, monocytes, plasmablasts, thrombocytes, and long-lived memory B cells) (11, 13, 148, 149). The splenic resident macrophages contain several subsets, such as red pulp macrophages (RPMs, VCAM-1^hi^ F4/80^+^ CD68^hi^), marginal zone macrophages (MZMs, SIGN-R1^+^, or SIGN-R1^-^), marginal metallophilic macrophages (MMMs, CD169^+^ MHC-II^+^ MOMA-1^+^), tingible body macrophages (TBMs, CD68^+^ MFG-E^+^ MerTK^+^ Tim4^+^ CD36^+^), and T zone macrophages (TZMs, MerTK^+^ CX3CR1^+^) (11, 149). They reside in the different locations of the spleen, with distinct developmental origins, phenotypes, and functions (11, 149) (**Table 1**).

**Table 1:** The classification and function of the PC–TRM crosstalk in tissue homeostasis.

Organ	PC	TRM	Crosstalk signals	Function	Ref.
CNS	Neuron	Microglia	Purines; chemokines; MMP-3; glutamate; TREM2; IgSF; IL-33–NF-κB; CD36; Intercellular Ca^2+^ wave (ICW) communication; TNFα; complement factors; CX3CL1–CX3CR1; TGFβ; CD22; VEGF; fractalkine; IGF1; TLR9	Synaptic pruning Axon fasciculation Promote neural precursor cell proliferation and survival Neurite formation Synaptogenesis	(28, 30, 34, 35)
Intestine	IEC	Intestinal macrophage	TRPV4; PGE2; HGF; WNT ligands; IL-4; CSF1; VEGF; BMP2	Gut motility Gastrointestinal secretion Dead cell clearance Epithelial homeostasis maintenance Immune sentinel functions Antimicrobial activity	(56, 57, 64, 66)
	Smooth muscle cell				
	Myenteric neuron				
Adipose tissue	Adipocyte	VAM	CD206; CD163; TGFβ; IL-1β	Regulation of complement system, blood vessel morphology, and endocytic capacities	(11, 73, 75, 78, 91)
		LAM	TNFα; chemokines; IL-1β; CD36; TREM2	Dead adipocytes and lipid clearance	
		ATM	Collagens; ADEs; Shh; IL‐6; PDGFcc	ECM deposition Tissue remodeling Adipocyte function modulation	
Heart	Cardiomyocyte	CM	MerTK; amphiregulin (AREG)	Clearance of infectious agents, cellular debris, and extracellular hazardous substances Maintenance of the cardiac electrical conduction	(126, 132)
Liver	Hepatocytes	KCs	IL-1β; PPARα; TNFα; NF-κB; IL-6; TREM2; microRNA; mtDNA	Clearance of erythrocytes and blood pathogens Iron metabolism Lipid metabolism Immunological tolerance	(95, 104, 110, 115, 119, 120, 150)
		LCMs		Immune surveillance Neutrophil recruitment	
Lung	Pulmonary epithelial cells	AMs	PPARγ; TGFβ; GM-CSF; lipid; cholesterol	Surfactant clearance Inhaled particles Phagocytosis Mediation of immune sentinel functions	(134, 142, 143, 145, 151)
		IMs	CD206, LYVE1, IL-10, MHC-II	Pathogens/infections Clearance Immune sentinel Mediation	

The crosstalk between RPMs and fibroblasts is shown in **Figure 1**. RPMs reside in the splenic cord and are closely associated with RP fibroblasts (152). PCs are a group of cells responsible for the primary functions of tissues. The spleen mainly acts as a blood filter (selectively removing circulating pathogens, dysfunctional red blood cells, and immune complexes), blood storage site, and blood volume regulator (13, 148). Unlike other tissues (those of the CNS, liver, lung, etc.), the distinction between PCs and supportive cells may still be unclear in the spleen.

The red pulp is composed of fibroblasts and reticular fibers that form a complex framework of open blood circulation, allowing for the selective removal of senescent and dysfunctional red blood cells (13). Therefore, from blood storage and blood volume regulation functions, fibroblasts are similar to PCs, and RPMs act as supportive cells. RPMs can support the survival, proliferation, and ECM secretion of RP fibroblasts *via* trophic factors (13, 148, 149). RPMs communicate with RP fibroblasts by expressing TGFβ and progranulin, and RP fibroblasts express TGFβ-RIII (a coreceptor for active TGFβ) and TNFRSF1A/B for survival (152, 153). RPMs also regulate the survival and proliferation of PDGFRα/β^+^ RP fibroblasts by producing PDGFβ (154, 155). In addition, RPMs can also regulate the reticular structure of RP through the production of proteases and the modulation of fibroblastic activity (11). Thus, RPMs are involved in controlling the quality of blood filtration indirectly.

Both immune defense and the maintenance of iron homeostasis are essential functions of the spleen, in which RPMs play a crucial role (152, 156). RPMs are PCs that function in blood filtration, whereas fibroblasts act as supportive cells and are critical regulators of macrophage homeostasis in RP. WT1^+^ reticular fibroblasts regulate the proliferation and location of RPMs through the production of CSF1 (152). Activation of the transcription factor Spi-C and heme oxygenase (HO)-1 is required for intracellular heme breakdown and free iron release from RPMs (156, 157). This molecular mechanism can neutralize the toxic effects of heme and metabolize iron (156, 157). Thus, RPMs can degrade the toxic cargo when senescent red blood cells are captured (156, 157).

Additionally, extramedullary hematopoiesis is supported by RPMs, and their absence impairs the recovery of normal red blood cell counts (152). Stress erythropoiesis causes the rapid production of mature erythrocytes. Previous research has indicated that RPMs can release a critical regulator (called GDF15) to expand the stress erythropoietic niches (158, 159). RPMs induce RP fibroblast-secreted BMP4 to maintain a suitable microenvironment and produce GDF15 to promote stress erythropoiesis in the spleen (158, 159).

However, since the spleen is a vital lymphoid organ for clearing blood pathogens, researchers mainly focus on the function of spleen macrophages in removing bacteria and regulating their interaction with other immune cells as well as the interaction between macrophages and fibroblasts in the spleen. The intercommunication between splenic macrophages and fibroblasts in splenic homeostasis and diseases is still unclear.

## Conclusions

With the development and escalation of sequencing techniques, the genetic landscapes of different tissues have been mapped and are continuously improved through single-cell transcriptome and spatial metabolomics (2, 99, 160–163). Information on PC‐NPC interactions is constantly being mined (99, 150, 151, 160–167). Research on the communication between them has found that the PC‐TRM crosstalk is instrumental in maintaining overall tissue homeostasis through cell membrane receptors, inflammatory or anti-inflammatory cytokines, metabolites, and extracellular vesicles (**Table 1**). Meizlish and colleagues put forward the following definition of tissue homeostasis: a collection of circuits regulating specific variables within the tissue microenvironment (166). The values of regulated variables are monitored by a controller (166). TRMs are homeostatic controllers that can monitor fluctuating environmental signals directly or indirectly and react in certain ways, such as pathogen clearance, apoptotic cell phagocytosis, ECM modification, and cytokine secretion (1, 166).

The functional demand can be deemed the deviation of a homeostatic variable, and signals are the proxies of homeostatic variations that report on practical demands (1). From this, we posit that PCs sense and reshape the functional demand during environmental fluctuations. While TRMs act as environmental “sensors” and “gatekeepers,” they have strong plasticity and motility for phenotype reshaping to respond to the variational signals of the environment and PCs. Macrophages can express certain substances through negative feedback signals and responsive PCs to bring the off-balance value back to its equilibrium point. For instance, neurons perform impulse conduction continuously and discharge “excess ATP” (40). However, the surrounding microglia sense the “excess ATP” and generate negative feedback signals to prevent neuronal overactivation (40). Furthermore, CMs sense and ingest cardiomyocyte-derived vesicles to avoid the accumulation of harmful extracellular substances (132). In the lung, the capture and metabolism of surfactants *via* AMs are critical for maintaining lung biomechanics (141, 142). In turn, pulmonary epithelial cells regulate macrophage phenotypes and functions through the WNT/β-catenin pathway and epithelial-derived S100A8/A9 (145). Splenic RP fibroblasts regulate the proliferation and location of RPMs through CSF1 to maintain iron homeostasis in the spleen, whereas RPMs can support the survival and proliferation of fibroblasts and regulate the functions of RP fibroblasts (152, 156–159).

The role of TRMs cannot be unilaterally defined as “good” or “bad” but depends on the signals from the microenvironment and peripheral cells. In KCs, FFA-induced NLRP3 inflammatory body activation promotes the production of proinflammatory IL-1β (119, 120). In contrast, KCs induced by IL-4/IL-13 produce M2-type exosomes and regulate insulin resistance of HCs through the miR-690–*Nadk* axis (106). Additionally, KC-derived TNFα has been confirmed as one of the inducers of HC steatosis (117). However, a recent study showed that KCs can regulate ketone generation in HCs during fasting and maintain hepatic and systemic metabolism (104). Similarly, the effect of macrophages on adipocyte metabolism is not one-fold. M2-like macrophages affect adipogenesis, and the heat production of adipocytes helps regulate energy storage in WAT to respond to energy needs (71, 80). Under pathological conditions, adipocytokines stimulate M1 macrophages, which aggravate insulin resistance, obesity-induced inflammation, and metabolic disorders (89). Among these, the adjustment mechanism of the threshold points of the transformation from “favorable” to “unfavorable” is still a “mystery.” Therefore, further investigations are necessary to clarify the “mutual benefit” or “mutual restraint” relationship between PCs and TRMs.

In recent years, bioinformatics techniques combined with transcriptome, proteomics, and spatial data have been widely used to predict intercellular communications and map cell space (2, 99, 160–163). Thus, we can obtain evidence for the heterogeneity of PCs and TRMs in various tissues and the differences in cell‐cell communication in different regions. Additionally, we should further understand the transformation or regulation mechanisms involved in communication under physiological and pathological conditions. With the gradual deepening of our understanding of “zonation,” it is worth exploring what regulatory mechanisms may exist for cell interactions between different anatomical regions in tissue in the future.

## Author contributions

YC and LT contributed to the conception of this work. YC drafted the manuscript and prepared figures using Adobe Illustrator 2021. LT supervised and edited the manuscript. All authors contributed to the article and approved the submitted version.

## Funding

This work was supported by the National Natural Science Foundation of China (Grant Nos. 31900632, 31900666, 82225009).

## Conflict of interest

The authors declare that the research was conducted in the absence of any commercial or financial relationships that could be construed as a potential conflict of interest.

## Publisher’s note

All claims expressed in this article are solely those of the authors and do not necessarily represent those of their affiliated organizations, or those of the publisher, the editors and the reviewers. Any product that may be evaluated in this article, or claim that may be made by its manufacturer, is not guaranteed or endorsed by the publisher.
